# Assessing the Resilience of sEMG Classifiers to Sensor Malfunction and Signal Saturation

**DOI:** 10.3390/s26082386

**Published:** 2026-04-13

**Authors:** Congyi Zhang, Dalin Zhou, Yinfeng Fang, Dongxu Gao, Zhaojie Ju

**Affiliations:** 1 School of Computing, Mathematics and Physics, University of Portsmouth, Portsmouth PO1 3HE, UK; congyi.zhang@myport.ac.uk (C.Z.); dalin.zhou@port.ac.uk (D.Z.); dongxu.gao@port.ac.uk (D.G.); 2 School of Communication Engineering, Hangzhou Dianzi University, Hangzhou 310005, China; yinfeng.fang@hdu.edu.cn

**Keywords:** surface electromyography, robustness evaluation, saturation (clipping), sensor dropout, time-domain features, random forest, real-time learning

## Abstract

Surface electromyography (sEMG) is widely used for gesture recognition, yet the way classic feature–classifier pipelines fail under realistic signal degradations is still poorly quantified. Existing studies typically report accuracy on clean laboratory data, leaving open how amplitude saturation and channel dropout jointly affect different feature combinations, classifiers, and subjects. In this work, we provide, to our knowledge, the first systematic robustness map of a conventional sEMG pipeline under controlledclipping and single-sensor failure. sEMG from nine subjects performing a multi-session, multi-gesture protocol is windowed (250 ms, 50 ms hop) and represented using four common time-domain features (Root Mean Square, Variance, Zero Crossing, and Waveform Length). We exhaustively evaluated single features and all pairwise fusions with three standard classifiers (Support Vector Machine (RBF kernel), Linear Discriminant Analysis, and Random Forest) over (i) a sweep of symmetric saturation thresholds ($10^{-6}$–$10^{-1}$) and (ii) five single-channel dropout scenarios, reporting subject-wise dispersion rather than aggregate scores alone. This design enables explicit characterization of the following: (1) accuracy recovery as clipping weakens for each feature pair; (2) dependency of robustness on which channel fails; and (3) differences among Support Vector Machine, Linear Discriminant Analysis, and Random Forest under identical degradations. The results show that lightweight feature pairs (Root Mean Square + Waveform Length, Variance + Zero Crossing, and Waveform Length + Zero Crossing) coupled with Random Forest form a consistently robust operating point, with performance recovering as clipping weakens and remaining resilient under single-channel dropout. Beyond robustness, the conventional pipeline trains substantially faster than representative deep learning baselines under a unified end-to-end timing definition, supporting real-time recalibration and repeated robustness sweeps in wearable deployments.

## 1. Introduction

Surface electromyography (sEMG) has become a fundamental sensing modality to decode human intention in neurorehabilitation, prosthetic control, and human-robot interaction [1]. Accurate recognition of upper-limb gestures from sEMG is critical for restoring functional movements in individuals with motor impairments and for enabling intuitive human-machine interfaces. Over three decades of research, from early time-domain pattern-recognition pipelines to modern deep-learning approaches, has yielded high accuracies in controlled laboratory settings [2,3]. However, reliable out-of-lab translation remains constrained by both robustness gaps and deployment constraints.

In wearable use, sEMG acquisition is affected by multiple sources of nonstationarity: inter-/intra-subject variability across days and sessions, muscle fatigue, electrode displacement during donning/doffing, changes in skin–electrode impedance due to sweat or temperature, cable motion artifacts/analog-to-digital converter (ADC), and power-line interference [4]. In addition, acquisition front-end saturation (clipping) occurs when instantaneous amplitudes exceed the dynamic range of the amplifier. These factors bias time-domain descriptors, e.g., Root Mean Square (RMS), Variance (VAR), Waveform Length (WL), and Zero Crossing (ZC), that underpin classic pipelines and degrade the separability learned by statistical classifiers [1].

Electrode shift and day-to-day variation are among the leading causes of performance drops in myoelectric control [5]. Recent studies have increasingly explored deep learning, domain adaptation, and noise-robust training strategies to mitigate inter-session and inter-subject variability in sEMG recognition. These approaches are promising for improving generalization under nonstationary conditions, but they often introduce higher computational cost, greater implementation complexity, and less transparent failure behavior under specific hardware-related degradations. In contrast, the present study focuses on a controlled robustness analysis of lightweight feature–classifier pipelines, with the aim of identifying practically deployable operating points under clipping and channel dropout. Remedies include channel selection and high-density montages, feature normalization and pairing strategies, adaptive/transfer learning [6], and controller-level fallback strategies (e.g., confidence-based rejection or recalibration). Meanwhile, nonparametric ensembles such as Random Forests (RFs) can offer favorable accuracy and robustness trade-offs under distribution shift compared with linear models or margin-based Support Vector Machines (SVMs) in several sEMG recognition settings [7]. Nevertheless, two practically common distortions are still under-characterized under a unified, controlled analysis: saturation-induced clipping of the time waveform and single-channel dropout due to intermittent electrode detachment. Both arise naturally in wearable scenarios; tight sleeves, sweat, or cable tug can push signals to rails or break contact. Both directly perturb the statistics estimated by lightweight features.

This study systematically quantifies how these distortions affect recognition across representative feature–classifier pairings. To control protocol variance while retaining session-to-session realism, each participant completes one session per day over three consecutive days (i.e., three sessions per subject). Within each session, randomized on-screen prompts cue 13 s gesture holds spanning functional daily motions (three hand postures, six wrist movements, and our grasp types) [8]. A structured rest schedule is used within each session (including longer breaks between gesture blocks) to reduce fatigue accumulation and maintain recording consistency. We then sweep symmetric amplitude bounds to emulate different clipping severities and simulate single-channel loss to emulate dropout [9].

We focus on four lightweight and widely used time-domain features (RMS, VAR, WL, and ZC) and their pairwise fusions, and compare three representative classifiers, RF, Linear Discriminant Analysis (LDA), and SVM, selected for complementary inductive biases and practical deployment cost [10,11]. Accuracies are reported window-wise using repeated stratified splits; aggregation across subjects is made explicit to distinguish central trends from inter-subject dispersion. This design supports two practical questions: (i) which feature pairs remain stable as clipping weakens/strengthens and (ii) which classifier sustains accuracy under single-channel loss. This choice was intentional, as the target scenario of this study is real-time wearable deployment, where low computational overhead, predictable latency, and rapid recalibration are important practical constraints.

Our contributions are threefold. First, rather than introducing a new classifier architecture, we establish a controlled robustness evaluation framework for wearable sEMG recognition under two practically relevant hardware-related degradations, namely, amplitude clipping and single-sensor dropout. Second, within this unified setting, we systematically quantify how representative lightweight feature–classifier combinations respond to degradation severity, identifying robust operating points and reporting cross-subject dispersion. Third, we complement the robustness analysis with a unified runtime evaluation, showing that conventional feature-based machine learning pipelines train substantially faster than representative deep learning baselines, which supports real-time recalibration and repeated stress testing. Together, these contributions provide actionable guidance for designing robust and lightweight sEMG systems for wearable, out-of-lab deployment.

## 2. Related Work

### 2.1. Sensing Modalities

#### 2.1.1. Electromyography

EMG captures the electrical manifestation produced by muscle activation [12]. Because EMG reflects neuromuscular activity associated with voluntary motion, it has been widely adopted for motor-function rehabilitation and replacement, including post-stroke assistive control and myoelectric prostheses [13,14]. EMG acquisition can be broadly categorized into intramuscular EMG (iEMG) and surface EMG (sEMG). iEMG provides high selectivity by inserting electrodes into muscle fibers, but its invasiveness limits user acceptance and practical deployment. In contrast, sEMG is noninvasive and easy to acquire, and thus, it remains the mainstream option for wearable interfaces despite increased susceptibility to cross-talk, motion artifacts, and contact instability [13,14]. These practical limitations motivate robustness-oriented evaluation beyond clean laboratory conditions.

#### 2.1.2. Electroencephalography

Electroencephalography (EEG) remains a widely used noninvasive brain-signal modality for brain–computer interfaces because of its portability and relatively low hardware cost [15]. However, robust intention decoding from wearable EEG is still challenging in real-world settings, where low signal-to-noise ratio, inter-session variability, and diverse artifacts can substantially degrade performance [16]. Recent evaluations further show that practical deployment typically requires artifact-aware processing, careful channel configuration, and robustness-oriented model design to balance accuracy, latency, and usability [17]. Although EEG provides complementary neural information compared with muscle-based sensing, its noise characteristics and temporal constraints remain substantially different from surface electromyography-based motor decoding.

#### 2.1.3. Ultrasound

Ultrasound has re-emerged as a promising noninvasive sensing modality for motion-intent decoding because it captures morphological muscle changes beyond superficial electrical activity and is inherently resistant to electromagnetic interference [18]. Recent sonomyography studies report strong performance for fine-grained upper-limb motion estimation and gesture-related tasks in controlled conditions [19]. Nevertheless, robustness in daily use is still limited by probe shift, coupling variation, and user-dependent placement effects, which can cause noticeable performance drops and necessitate recalibration strategies.

#### 2.1.4. Force Myography

Force myography measures pressure/tension changes at the skin surface caused by underlying muscle contractions and has shown practical potential for wearable gesture interfaces with low-cost hardware [20]. Recent reviews indicate that force myography can provide reliable control cues in many scenarios, but sensitivity to strap tightness, sensor placement, and soft-tissue mechanics remains a key challenge for cross-user and cross-session generalization [21]. As a result, force myography is often best viewed as a complementary modality, particularly when subtle intention decoding or severe impairment requires richer physiological information.

#### 2.1.5. Others

Multimodal fusion has been adopted to mitigate the effects of adverse factors such as electrode/probe shift and physiological changes (e.g., sweating and fatigue). For example, near-infrared sensing can complement EMG by providing metabolic/oxygenation cues associated with fatigue, potentially improving robustness during long-term use [22]. Another common combination integrates sEMG with inertial measurement units (IMUs), which provide orientation and motion dynamics that can partially compensate for signal-quality degradation in sEMG [23]. While multimodal systems are promising, they also increase hardware complexity and may not be available in minimal wearable designs; thus, a principled understanding of robustness for classic single-modality pipelines remains valuable.

### 2.2. Sensory Data Processing

#### 2.2.1. Direct Recognition and Control

Despite extensive research demonstrating high accuracies for pattern-recognition-based control under laboratory conditions, many commercial rehabilitative devices still rely on direct EMG control strategies. Direct control maps sEMG amplitude to proportional commands for individual degrees of freedom, enabling intuitive control without predefined motion templates. However, simultaneous multi-degree-of-freedom (DOF) control is often clinically viable only after targeted surgical interventions such as muscular reinnervation, and practical performance can be limited by noise, variability, and insufficient separability when many gestures are required.

#### 2.2.2. Classic Pattern Recognition

Classic pattern recognition typically consists of windowing, feature extraction, and classification. Feature extraction converts raw signals into lower-dimensional descriptors intended to capture discriminative information while suppressing noise. Time-domain and frequency-domain features have been evaluated extensively, including nonlinear measures such as sample entropy, which can achieve strong accuracy and stability in certain settings. LDA is widely used because of simplicity and computational efficiency, and electrode configuration optimization can further improve recognition performance. These pipelines remain attractive for embedded deployment due to predictable latency, low memory, and fast retraining.

#### 2.2.3. Deep Learning Models

Deep learning models, including convolutional neural networks (CNNs) and recurrent neural networks (RNNs), can learn hierarchical representations from raw sEMG and have been applied to gesture recognition, joint angle prediction, and force estimation. CNNs are effective when sEMG is encoded as an “EMG image” that captures spatial dependencies, whereas RNN variants model temporal dynamics. Deep transfer learning and domain adaptation have been used to mitigate inter-session and inter-subject variability, and multimodal deep fusion (e.g., sEMG + IMU) can further improve robustness. However, deep models often entail higher training cost, hyperparameter sensitivity, and potentially longer recalibration time, which can be restrictive for real-time wearable workflows that require repeated retraining (e.g., after donning/doffing).

More recent studies have further explored robustness-oriented deep learning strategies for sEMG recognition, including transfer learning, domain adaptation, and self-supervised representation learning. These approaches aim to mitigate performance degradation caused by session shift, subject variability, and reduced label availability by learning more transferable latent representations or adapting models to target domains. For example, recent work has investigated transfer-learning-based multi-scale CNN models with domain adaptation and self-supervision for improved inter-subject generalization [24], recursive domain-adversarial learning to address day-to-day EMG variation [25], and calibration-free frameworks that combine self-supervised pretraining with adversarial domain alignment for subject-invariant sEMG intention recognition [26]. Such developments highlight an important trend in the field: improving robustness not only through stronger classifiers but also through better representation learning and adaptation mechanisms. However, these methods often introduce higher training cost, increased architectural complexity, and less transparent failure behavior under specific hardware-related degradations. For this reason, controlled robustness analyses of lightweight feature–classifier pipelines remain valuable, particularly when the target scenario emphasizes real-time recalibration, low computational overhead, and interpretable performance under clipping and channel dropout.

### 2.3. Applications

#### 2.3.1. Motor Function Replacement

Physiological-signal-driven prosthetic systems depend on accurate intention decoding. sEMG remains the most practical modality for prosthetic control due to accessibility on the residual limb and strong correlation with muscle activation [27,28]. Advances in feature engineering, adaptive learning, and deep learning have enabled increased dexterity and improved user-specific performance [29]. Modern devices increasingly support multi-DOF control and more naturalistic motion, but robustness to daily signal disturbances remains a key barrier.

#### 2.3.2. Motor Function Rehabilitation

Wearable exoskeleton robots and assistive devices provide restorative support for users with partial motor-function loss [30]. Integration with functional electrical stimulation (FES) can promote active engagement and improved outcomes [31]. As systems transition toward home-based rehabilitation, robustness to real-world sensing degradations becomes essential [32,33].

#### 2.3.3. Gap Between Research and Application

A persistent gap exists between high laboratory accuracies and reliable daily-life usability. The forearm is a convenient acquisition site because its muscle groups coordinate diverse hand and wrist motions, which supports gesture classification and assistive control. Yet, daily activities introduce variability (placement, impedance, and motion artifacts) that can induce clipping or dropout and degrade feature statistics, motivating controlled robustness evaluation under these distortions.

### 2.4. Signal Distortions in sEMG: Saturation and Channel Dropout

#### 2.4.1. Saturation (Clipping): Causes and Impact on Signal Fidelity

sEMG saturation (clipping) arises when recorded amplitudes exceed the dynamic range of the front-end or ADC. This can result from strong contraction, excessive gain, or suboptimal electrode–skin contact. Clipping truncates waveform peaks and valleys, distorting both time-domain features (e.g., RMS, WL) and frequency-domain metrics, and it can reduce effective information content available for classification.

#### 2.4.2. Channel Dropout (Electrode Disconnection): Mechanisms and Consequences

Channel dropout may manifest as flat-line segments, zero-value spans, or unstable bursts due to intermittent contact, electrode detachment, or cable movement. Such discontinuities corrupt temporal structure and can bias feature estimates, particularly when features assume continuous motor unit action potential activity.

**Figure 1:**
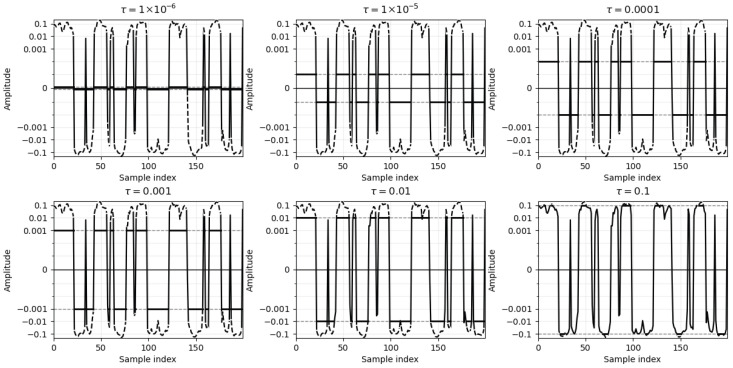
Saturation (clipping) under different thresholds. The same 200-sample segment is displayed for $\tau \in \{10^{-6},10^{-5},10^{-4},10^{-3},10^{-2},10^{-1}\}$. Within-range samples are plotted as continuous solid black lines; out-of-range portions of the original signal are shown as dashed black lines, and the clipped output is drawn as solid segments on the rails at ±τ (rails shown as gray dashed lines). The *y*-axis uses symmetric logarithmic ticks at ±10^−1^, ±10^−2^, ±10^−3^, and 0, centered at zero.

As illustrated in Figure 1, applying symmetric rails at ±τ to the same 200-sample segment renders out-of-range portions dashed (original), while the clipped output lies on the rails as solid segments. A smaller τ produces longer plateaus; as τ increases, rail hits diminish and feature bias reduces.

#### 2.4.3. Illustrative Example of Saturation Distortion

To make the saturation model concrete, we visualize in Figure 1 how clipping distorts a real sEMG waveform. Let x[n] denote a cleaned, normalized sEMG sample at discrete time index *n*, and let τ>0 be a symmetric amplitude bound. Saturation is modeled by the pointwise operator

(1) $$y\left[n\right]=C_{\tau }\;\left(x[n]\right)=\mathrm{sign}\;\left(x[n]\right)\;\min\;\left(|x[n]|,\tau \right),$$

which limits the observed signal y[n] to [−τ,τ] while preserving polarity. For Figure 1, we randomly draw a single analysis window from the preprocessed recordings of one subject (same window length as in subsequent feature extraction) and treat it as the reference waveform x[n]. The dashed curve in each panel shows the original segment, whereas the solid curve shows the clipped signal y[n] produced by (1) for $\tau \in \{10^{-6},10^{-5},10^{-4},10^{-3},10^{-2},10^{-1}\}$. For very small τ, most samples saturate to ±τ, primarily leaving sign structure; as τ increases, fewer samples satisfy |x[n]|>τ, and y[n] converges toward x[n]. This example is generated from recorded data (not synthetic) and illustrates the gradual recovery of amplitude information as clipping weakens.

## 3. Methodology

### 3.1. Data Preprocessing and Windowing

Surface EMG was recorded using a five-channel Delsys Trigno Avanti acquisition setup. Four channels were positioned around the forearm to cover the upper, lower, left, and right sides of the forearm, while an additional channel was placed over the biceps region. The Trigno Avanti sensors use a fixed inter-electrode distance of 10 mm and 16-bit resolution, with an EMG input range of approximately 11 mV. Signals were sampled at 1925.9259 Hz. This acquisition configuration is reported here to clarify the practical interpretation of the clipping threshold sweep and the realism of the single-channel dropout simulations used in this study.

During preprocessing, power-line interference was suppressed using notch filtering at 60 Hz and its harmonics, consistent with the local mains frequency, followed by Butterworth filtering before segmentation. The cleaned sEMG stream was segmented into overlapping windows of 250 ms with a hop of 50 ms (80% overlap). The 250 ms window with 50 ms hop was selected as a practical compromise between temporal responsiveness and feature stability. This configuration is widely used in sEMG pattern recognition because it preserves sufficient signal content for reliable feature estimation while maintaining latency compatible with real-time wearable interaction.

### 3.2. Handcrafted Time-Domain Features

We extract lightweight time-domain descriptors from each window to characterize amplitude, variability, complexity, and sign dynamics. Let $x_{i}$ denote the *i*-th sample in a window of length *N*. The RMS amplitude is

(2) $$\mathrm{RMS}=\sqrt{\frac{1}{N}\sum_{i=1}^{N}x_{i}^{2}}.$$

Variance is computed as

(3) $$\mathrm{VAR}=\frac{1}{N-1}\sum_{i=1}^{N}{\left(x_{i}-\bar{x}\right)}^{2},\;\bar{x}=\frac{1}{N}\sum_{i=1}^{N}x_{i},$$

and Zero Crossings count polarity changes between adjacent samples:

(4) $$\mathrm{ZC}=\sum_{i=1}^{N-1}\mathrm{sgn}\;\left(-x_{i}x_{i+1}\right),$$

with

(5) $$\mathrm{sgn}\left(x\right)=\left\{\begin{array}{cc} 1, & x>\varepsilon ,\\ 0, & x\leq \varepsilon , \end{array}\right.$$

where ε avoids counting noise-induced crossings. The Waveform Length is

(6) $$\mathrm{WL}=\sum_{i=1}^{N-1}\left|x_{i+1}-x_{i}\right|.$$

These features are computationally efficient and widely used in myoelectric pattern recognition, making them appropriate for real-time wearable settings.

### 3.3. Frequency-Domain Features

To broaden the feature comparison beyond the original lightweight time-domain baseline, an auxiliary experiment further considered four additional descriptors, namely, autoregressive coefficients (ARs), sample entropy (SAEN), median frequency (MDF), and mean frequency (MNF). Among these, MDF and MNF are frequency-domain descriptors derived from the estimated power spectrum of each sEMG window.

For a power spectral density estimate P(f) over frequency *f*, the mean frequency (MNF) is defined as

(7) $$\mathrm{MNF}=\frac{\sum_{f}f\;P\left(f\right)}{\sum_{f}P\left(f\right)},$$

which represents the spectral centroid of the window. The median frequency (MDF) is defined as the frequency that divides the spectral power into two equal halves:

(8) $$\sum_{f=0}^{\mathrm{MDF}}P\left(f\right)=\frac{1}{2}\sum_{f}P\left(f\right).$$

These frequency-domain features provide information complementary to amplitude- and sign-based time-domain descriptors, such as RMS, VAR, WL, and ZC. In particular, they characterize the distribution of spectral energy rather than waveform amplitude directly. Because MDF and MNF depend on spectral estimation, their behavior may differ from that of the original lightweight time-domain features under signal degradation, preprocessing variation, and window selection. Therefore, these descriptors were introduced as additional comparison features rather than as replacements for the original baseline. In the extended experiment, representative time-domain features were paired with frequency-domain features under the same classifier and evaluation settings to examine whether spectral information improves robustness under the intra-session and inter-session evaluation settings.

### 3.4. Intra-Session and Inter-Session Evaluation Protocols

To further examine how session variability affects the reported robustness, two supplementary evaluation settings were considered in addition to the main repeated-split analysis: intra-session and inter-session evaluation. In the intra-session setting, training and testing data were drawn from the same recording session such that the model was evaluated under relatively stable acquisition conditions. In the inter-session setting, training and testing were separated across sessions, thereby introducing session-to-session variation caused by repeated donning/doffing, electrode repositioning, and other practical sources of nonstationarity.

The purpose of this comparison is to determine whether the relative ranking of lightweight feature combinations remains consistent when the evaluation protocol moves from a within-session setting to a more challenging cross-session setting. In this way, the analysis provides a more deployment-relevant view of robustness by explicitly examining the effect of session shift on classification performance.

### 3.5. Dataset and Experimental Protocol

#### 3.5.1. Gesture Collection (3 Sessions)

Each participant completed one session per day over three consecutive days (i.e., 3 sessions per subject). During each session, subjects followed randomized gesture prompts displayed on a screen. Each prompt lasted 10 s, during which the subject maintained the indicated gesture. The gesture set comprised three basic hand postures—Hand Rest (HR), Hand Open (HO), and Hand Closed (HC); six wrist movements—Wrist Flexion (WF), Wrist Extension (WE), Wrist Pronation (WP), Wrist Supination (WS), Ulnar Flexion (UF), and Radial Flexion (RF); and four grasp types—Fine Pinch (FP), Key Pinch (KP), Spherical Grasp (SG), and Cylindrical Grasp (CG). To reduce fatigue accumulation and stabilize acquisition, a structured rest schedule was used within each session, including longer breaks between gesture blocks.

In total, the dataset includes sEMG recordings from nine subjects, each contributing 3 sessions, with one trial per gesture in each session.

#### 3.5.2. Data Processing and Normalization

Signals are segmented with the sliding window approach described above. For each windowed segment, RMS, VAR, ZC, and WL are extracted from each channel. Prior to feature extraction and clipping simulation, each sEMG channel is standardized using z-score normalization to preserve polarity:

$$x^{\prime }\left[n\right]=\frac{x[n]-\mu }{\sigma +\delta },$$

where μ and σ are computed from the training partition of each split and then applied to the corresponding validation/test data (with small δ to avoid division by zero). This reduces amplitude scale differences while preserving the sign structure required by ZC and the symmetric clipping model in (1).

#### 3.5.3. Classification and Evaluation Protocol

We evaluate three conventional classifiers: SVM (RBF kernel), LDA, and RF. Each classifier is trained and tested using single features and pairwise feature combinations to quantify the effect of feature fusion.

For each subject, we adopt 10 repeated stratified splits of the windowed dataset into 70%/15%/15% training/validation/test partitions, preserving class proportions. Hyperparameters are tuned on the validation set (grid search within each split), and performance is reported on the held-out test set. This yields 10 window-wise test accuracies per subject, classifier, and feature combination, which are averaged to obtain per-subject means. In the Results, we either aggregate per-subject means across nine subjects and report the overall mean with subject-wise min–max ranges or display per-subject means explicitly as indicated by the figure captions. All experiments were conducted in Python 3.9.15 using scikit-learn. No additional class rebalancing procedure was applied, because class proportions were preserved through stratified partitioning, and the experimental protocol was designed to maintain approximately balanced gesture samples across sessions.

### 3.6. Training Efficiency for Real-Time Deployment

Our target application is real-time wearable sEMG interaction. In this setting, models are not trained once and kept fixed; instead, they are frequently re-calibrated across sessions, after electrode repositioning, or after signal-quality shifts (e.g., clipping and channel dropout). Consequently, computational efficiency is a deployment requirement: slower retraining increases user waiting time, reduces usability, and limits the number of robustness sweeps feasible during development and validation.

To make timing comparable and reproducible, runtime is measured end-to-end using the same repeated-split protocol as the accuracy experiments. The reported total time includes the following:

- Data I/O + Windowing: Loading files and generating 250 ms windows with 50 ms hop;
- Feature Computation: RMS/VAR/ZC/WL extraction and feature assembly for conventional pipelines;
- Model Training: Split-wise fitting (epoch-based optimization for deep models);
- Evaluation: Test-time inference and metric aggregation.

**Figure 2:**
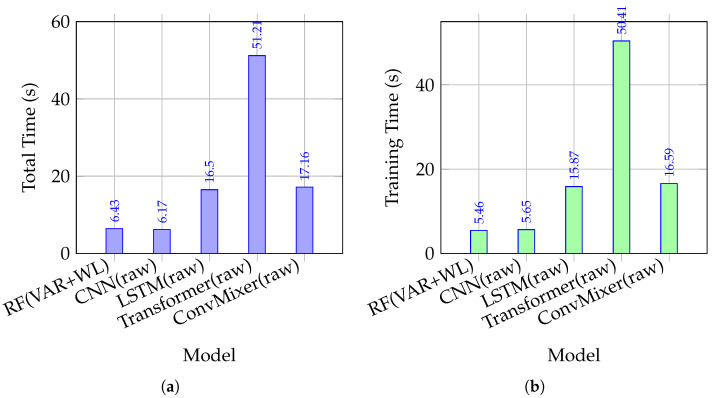
Runtime comparison between the proposed conventional pipeline and deep learning baselines under the same evaluation protocol. RF (VAR + WL) achieves a clear computational advantage while remaining robust under clipping and channel dropout. (**a**) End-to-end total runtime (including data I/O, windowing, feature computation when applicable, training, and evaluation). (**b**) Training runtime only (split-wise model fitting; deep models include epoch optimization).

Figure 2 presents the runtime comparison between the proposed conventional pipeline and the deep learning baselines under the same evaluation protocol.

Under this unified definition, we focus on training runtime as the primary metric, since it directly reflects the computational cost of model updating, robustness sweeps, and system recalibration in practical deployment. RF (VAR + WL) (CPU, scikit-learn) requires 5.46 s training time.

In contrast, deep models trained on raw input (GPU, PyTorch 2.2.2) exhibit higher training cost: CNN (raw) at 5.65 s, LSTM (raw) at 15.87 s, Transformer (raw) at 50.41 s, and ConvMixer (raw) at 16.59 s. Consequently, compared with CNN, LSTM, Transformer, and ConvMixer, RF (VAR + WL) reduces training runtime by approximately 3.36%, 65.60%, 89.17%, and 67.09%, respectively. These results demonstrate that the proposed conventional pipeline is highly efficient in terms of model training, particularly under repeated retraining scenarios, such as robustness evaluation and session-to-session recalibration. This efficiency, combined with its strong robustness under clipping and sensor dropout, makes RF (VAR + WL) especially suitable for resource-constrained wearable systems.

While the above results highlight the computational efficiency of the proposed RF (VAR + WL) pipeline, it is also important to examine whether more recent deep learning architectures can offer superior performance. To improve the interpretability of the runtime comparison, the deep learning baselines were implemented under the same raw-input evaluation framework. The CNN baseline consists of stacked one-dimensional convolution blocks followed by a fully connected classifier. The LSTM baseline applies recurrent sequence modeling to the input windows, and the Transformer baseline uses self-attention-based sequence encoding before classification. In addition, a lightweight ConvMixer architecture was included as a representative modern deep model, with an embedding dimension of 128, depth of 6, patch size of 10, and kernel size of 7.

All deep models were trained in PyTorch using the Adam optimizer with a learning rate of $10^{-3}$, batch size of 128, and 10 training epochs. The same cross-validation framework was used for timing and accuracy evaluation so that runtime should be interpreted together with the corresponding classification performance rather than as an isolated speed-only benchmark.

Under this setting, the conventional RF (VAR + WL) pipeline remains substantially more efficient in training time than the deeper sequence models, while ConvMixer provides the strongest deep-learning accuracy among the evaluated baselines. In particular, ConvMixer achieves a classification accuracy of 0.908, indicating that deep models still have the potential to improve recognition performance. However, this gain is accompanied by noticeably higher computational cost than the conventional RF-based pipeline. Therefore, under repeated retraining scenarios such as robustness sweeps, recalibration after reattachment, and real-time wearable deployment, the proposed RF (VAR + WL) pipeline provides a more favorable balance between robustness, training efficiency, and practical usability.

In the context of this study, where repeated robustness evaluation, rapid model updating, and real-time wearable deployment are key requirements, training efficiency becomes a critical constraint. Models must support fast retraining and low-latency operation under changing signal conditions (e.g., clipping and sensor dropout), which limits the practicality of computationally intensive deep architectures. Therefore, although ConvMixer demonstrates promising accuracy, the proposed RF (VAR + WL) pipeline provides a more suitable balance between performance, efficiency, and deployability for real-world applications.

## 4. Experimental Results and Discussion

### 4.1. Robustness Under Saturation (Clipping)

**Figure 3:**
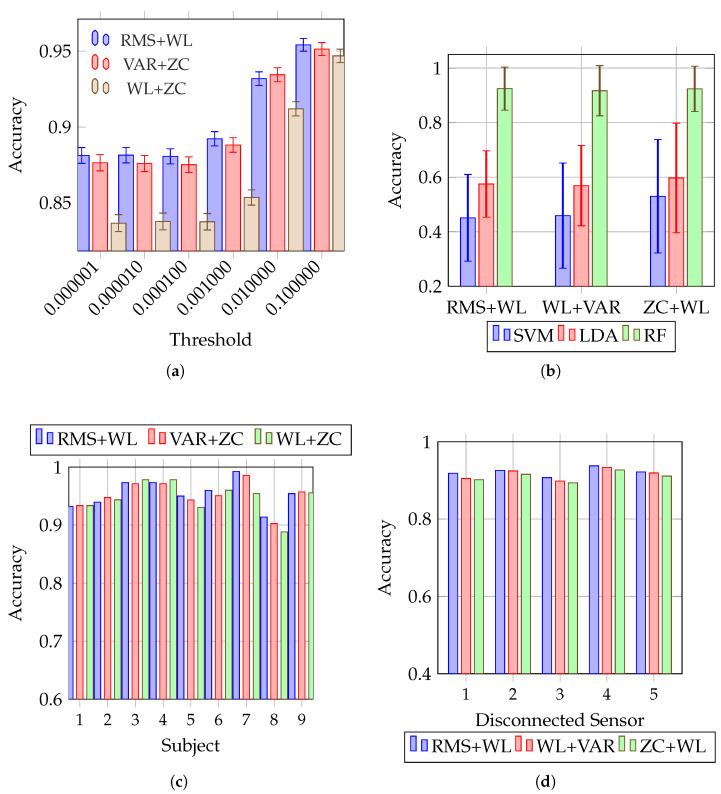
Robustness analysis under clipping and single-sensor dropout, together with classifier comparison and subject-wise consistency for the selected feature pairs. (**a**) RF accuracy across saturation thresholds for three feature pairs (RMS + WL, VAR + ZC, WL + ZC). Bars: Mean test accuracy across 9 subjects (per-subject mean over 10 splits); I-bars: subject-wise min–max. (**b**) Accuracy of SVM, LDA, and RF on three feature pairs. Bars: Mean test accuracy across 9 subjects (per-subject mean over 10 splits); I-bars: subject-wise min–max. (**c**) Subject-wise RF accuracy for three feature pairs. Each bar: Mean test accuracy of one subject over 10 splits. (**d**) RF accuracy under single-sensor disconnection (channels 1–5) for three feature pairs. Bars: Mean test accuracy across 9 subjects (per-subject mean over 10 splits).

This subsection examines how classification accuracy changes with clipping severity. Figure 3a reports RF accuracy across six saturation thresholds for the three strongest feature pairs. Bars show the mean test accuracy across nine subjects (each subject contributes a mean over 10 repeated splits), and I-shaped error bars indicate the subject-wise min–max range. Across all three pairs, accuracy increases monotonically with τ, with performance approaching or exceeding 0.95 at $\tau =10^{-1}$. This trend is consistent with the intuition that as clipping weakens, the distortion introduced by (1) diminishes and feature statistics better approximate their unclipped values.

From a signal perspective, increasing τ reduces the probability of saturation events. Under a zero-mean Gaussian approximation $x∼N(0,\sigma^{2})$, the saturation probability decreases with τ as

$$P_{\mathrm{sat}}=\mathrm{Pr}\;\left(|x|>\tau \right)=2\;Q\left(\frac{\tau }{\sigma }\right),$$

where Q(·) is the Gaussian tail probability. Because clipping replaces out-of-range samples by ±τ, it reduces signal energy in a threshold-dependent manner, implying RMS(y)≤RMS(x) and biasing amplitude-sensitive time-domain features. As τ increases, the fraction of clipped samples decreases and the bias in RMS/VAR/WL diminishes, providing a mechanistic explanation for the observed recovery of classification accuracy.

Importantly, the error bars contract at higher thresholds, indicating that subject-wise dispersion tends to reduce as clipping weakens. This is practically relevant: in wearable deployments, moderate reductions in clipping severity (e.g., through proper gain setting) can improve both the mean performance and reliability across users.

### 4.2. Classifier Dependence Under Identical Features

This subsection examines whether classifier choice remains an important factor when the same feature pairs are used. Figure 3b compares SVM, LDA, and RF on the same feature pairs, with subject-wise min–max dispersion. RF substantially outperforms SVM and LDA across all three combinations, indicating that classifier choice remains a dominant factor even when features are fixed. The wide dispersion observed for SVM and, to a lesser extent, LDA suggests that their performance is more sensitive to inter-subject variability and/or distorted feature distributions under the evaluated stress conditions. In contrast, RF appears to provide a more stable decision rule under the same inputs, consistent with its ensemble-based robustness to feature perturbations and nonlinear class boundaries.

### 4.3. Subject-Wise Consistency

This subsection examines the consistency of RF performance across individual subjects for the strongest feature pairs. Figure 3c provides subject-wise RF accuracies for the three feature pairs. While minor variations are present, all three combinations maintain high accuracy across subjects, with only modest degradation for the lowest-performing subject. This observation supports the use of pairwise time-domain feature fusion as a practical compromise between single-feature simplicity and higher-dimensional feature stacks that may increase sensitivity to channel quality issues.

### 4.4. Robustness Under Single-Channel Dropout

This subsection examines how robust the selected RF feature pairs remain when a single sensor is disconnected. Figure 3d summarizes performance under single-sensor disconnection (channels 1–5). Even when one channel is removed, all three feature pairs retain accuracies above approximately 0.89. RMS + WL yields the highest and most stable performance across dropout cases, suggesting that this pair preserves complementary information that remains informative despite partial channel loss. Differences among dropout cases also indicate that robustness can depend on which channel fails, consistent with channel-specific informativeness due to placement and muscle recruitment patterns.

### 4.5. Comparison Between Intra-Session and Inter-Session Performance

**Figure 4:**
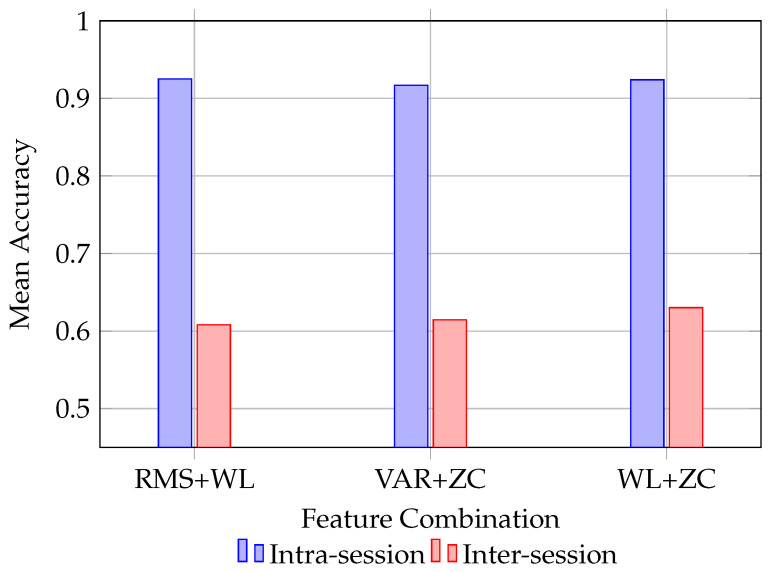
Comparison between intra-session and inter-session evaluation for three representative time-domain feature pairs. The inter-session setting yields lower but more conservative performance estimates, reflecting the additional challenge introduced by session-to-session variability.

Figure 4 compares the performance of three representative time-domain feature pairs under intra-session and inter-session evaluation settings. As expected, the inter-session condition yields consistently lower accuracy than the intra-session condition, indicating that session-to-session variability remains a substantial source of performance degradation in wearable sEMG recognition.

Importantly, the comparison shows that although the absolute performance level decreases under inter-session evaluation, the relative behavior of the stronger feature pairs remains broadly consistent. In other words, the more robust combinations continue to rank favorably even when the task becomes more challenging due to session shift. This finding is practically relevant, because wearable systems are repeatedly reattached across sessions, and therefore, robustness under inter-session variability is more indicative of real deployment performance than within-session accuracy alone.

### 4.6. Effect of Adding Frequency-Domain Information

**Figure 5:**
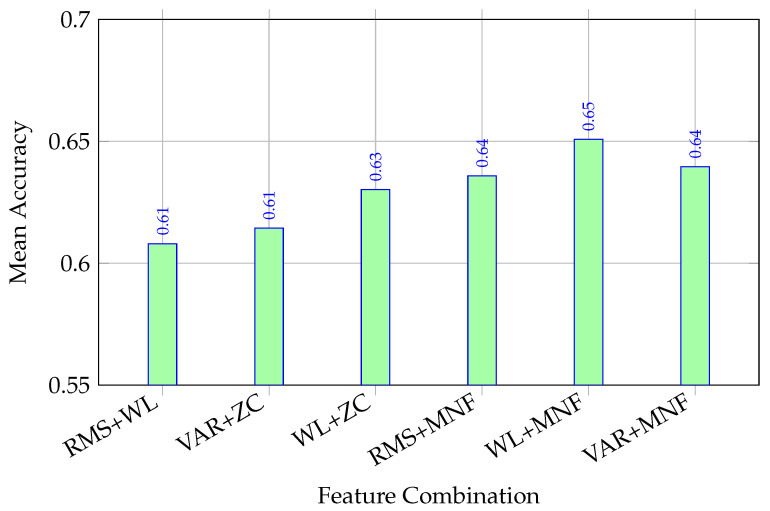
Comparison of representative time-domain-only pairs and extended time–frequency pairs. Bars show the mean accuracy across nine subjects. The inclusion of MNF changes the ranking of the strongest feature pairs, with WL + MNF yielding the highest mean accuracy among the tested combinations.

Figure 5 compares three representative time-domain-only pairs (RMS + WL, VAR + ZC, and WL + ZC) with three extended pairs that incorporate MNF under the inter-session evaluation setting. A clear change in ranking is observed after the inclusion of the frequency-domain descriptor. Among all tested pairs, WL + MNF achieves the highest mean accuracy (0.6508), followed by RMS + MNF (0.6358) and VAR + MNF (0.6396), whereas the strongest time-domain-only pair, WL + ZC, reaches a lower mean accuracy of 0.6302.

This result suggests that MNF provides complementary discriminative information beyond purely time-domain amplitude- and sign-based descriptors. At the same time, MNF should not be interpreted as a universally more stable descriptor. Unlike RMS, VAR, WL, and ZC, which are computed directly from waveform amplitude and sign transitions, MNF depends on spectral estimation and is therefore influenced by the quality of the estimated power spectrum. Previous tutorials and reviews on sEMG frequency analysis note that spectral features are affected by signal stationarity, epoch selection, filtering, and noise contamination and that both time- and frequency-domain characteristics can vary with electrode placement and physiological conditions.

Therefore, the present findings are interpreted more narrowly: Under the revised partitioning protocol used here, adding MNF improves the strongest feature pairs, but this does not imply that frequency-domain descriptors are intrinsically more robust in all wearable settings. Rather, the results indicate that MNF can provide useful complementary information when combined with lightweight time-domain features, while its behavior should still be considered in relation to preprocessing, window definition, and signal quality.

For clarity, the main numerical comparisons from the supplementary analyses are summarized in Table 1.

The summary confirms two consistent observations: first, inter-session evaluation is more challenging than intra-session evaluation for the same time-domain pairs; second, under the stricter inter-session setting, MNF-based pairs outperform the original time-domain-only combinations.

### 4.7. Summary of Findings in the Context of Wearable Deployment

Taken together, Figure 3 provides converging evidence that RF paired with lightweight time-domain feature fusion yields a robust operating point under two common wearable degradations: saturation (clipping) and single-channel dropout. When combined with the training-time advantages quantified in Figure 2, the conventional pipeline supports real-time usage modes that require repeated recalibration (e.g., after donning/doffing) and systematic stress testing during development. In this sense, robustness and computational efficiency are not competing objectives in the proposed configuration; rather, they jointly support practical deployment constraints.

### 4.8. Limitations

A limitation of this study is the relatively small dataset size, which may restrict the generalizability of the findings. Therefore, the present results should be interpreted primarily as evidence of relative robustness trends within a controlled experimental setting rather than as definitive population-level conclusions. Future work will extend the evaluation to larger cohorts and broader validation settings.

## 5. Future Work

This study demonstrates that simple time-domain feature fusions (Root Mean Square + Waveform Length, Variance + Zero Crossing, Waveform Length + Zero Crossing) paired with Random Forest yield robust performance under amplitude saturation and single-channel dropout. We will add saturation-aware preprocessing with winsorization, trimmed statistics, stochastic saturation-augmentation, and asymmetric/dynamic clipping; test k-of-5 and bursty/intermittent losses using feature masking or lightweight imputation, quantifying accuracy–variance trade-offs; compare lightweight temporal convolutional networks, one-dimensional convolution, and attention blocks against Random Forest, Support Vector Machine, and Linear Discriminant Analysis on accuracy, latency, and memory; pursue few-shot and streaming adaptation with drift detection and calibrated confidence-based abstention; and validate on wearable hardware by profiling accuracy, latency, and energy and releasing stress-test scripts with significance testing. Although the present study focuses on controlled clipping and single-sensor dropout, real wearable systems may also experience more complex degradation patterns. Future work will therefore extend the current framework to multiple simultaneous sensor failures, intermittent or bursty dropout, and external noise interference.

## Figures and Tables

**Table 1:** Summary of the main comparative results under the supplementary evaluation settings. The table combines the intra-/inter-session comparison for representative time-domain pairs and the extended inter-session comparison including MNF-based pairs.

Feature Pair	Intra-Session	Inter-Session	Feature Domain
RMS + WL	0.9250	0.6080	Time domain
VAR + ZC	0.9170	0.6144	Time domain
WL + ZC	0.9240	0.6302	Time domain
RMS + MNF	–	0.6358	Time + frequency
WL + MNF	–	0.6508	Time + frequency
VAR + MNF	–	0.6396	Time + frequency

## Data Availability

No new data were created or analyzed in this study.
